# A novel agricultural commodity price prediction model integrating deep learning and enhanced swarm intelligence algorithm

**DOI:** 10.1371/journal.pone.0337103

**Published:** 2025-12-02

**Authors:** Kaixuan Sun, Qi Yao, Yanhui Li

**Affiliations:** 1 School of Economics and Management, Huainan Normal University, Huainan, China; 2 School of Information Management, Central China Normal University, Wuhan, China; 3 Management School, Wuhan College, Wuhan, China; National Institute of Technology Rourkela, INDIA

## Abstract

The volatility of agricultural commodity prices significantly affects market stability and financial market dynamics, especially during periods of economic uncertainty and global shocks. Accurate price prediction, however, remains challenging due to the complex, nonlinear characteristics of agricultural markets and the diverse range of influencing factors. To overcome these challenges, this study develops a novel price forecasting framework that combines advanced time series decomposition, swarm intelligence optimization, and deep learning techniques. The proposed framework employs successive variational mode decomposition (SVMD) to deconstruct the raw price data into multiple components, effectively capturing the underlying nonlinear patterns and dynamic features. These components are then fed into a CNN-augmented BiLSTM model, enhanced with an attention mechanism to extract both temporal dependencies and intricate data relationships. To fine-tune the model’s hyperparameters, this study introduces a multiple strategies dung beetle optimisation algorithm (MSDBO), which integrates four strategic modifications to improve the balance between global search, local exploration, and convergence efficiency. Using historical data from corn and wheat markets as case studies, the experimental findings demonstrate that the proposed SVMD-MSDBO-CNN-BiLSTM-A model significantly outperforms nine baseline approaches. Specifically, it reduces the Mean Absolute Percentage Error (MAPE) by 25.78% and 37.57%, respectively, and enhances directional accuracy (*D*_*stat*_) by 1.15% and 14.53% compared to the top single models.

## 1 Introduction

The volatility of agricultural commodity prices is a critical aspect of global financial market dynamics and economic stability. Agricultural commodities, as key components of the broader commodity markets, play an essential role in ensuring food security, supporting economic growth, and facilitating global trade. However, the price movements of these commodities are inherently nonlinear, driven by factors such as macroeconomic shocks, geopolitical tensions, supply-demand imbalances, and climate variability [1–3]. These complexities have been exacerbated by escalating global trade tensions and supply chain vulnerabilities, which have altered traditional price transmission mechanisms and introduced new variables into price formation processes [4,5]. Moreover, given their close integration with financial systems, disruptions in agricultural commodity prices can generate spillover effects, amplifying risks for investors, policymakers, and market regulators [6].

Recent global events, including health emergencies, inflationary pressures, and geopolitical risks, have further complicated price dynamics [7]. These events have heightened market uncertainty and volatility, underscoring the urgent need for robust, data-driven forecasting frameworks capable of capturing the nonlinear characteristics of agricultural commodity prices. Such models are critical for improving risk management, financial market regulation, and understanding price dynamics under volatile conditions [8,9].

Nevertheless, predicting agricultural futures prices remains a highly complex task. These include economic globalization, climate variability, and financial speculation, each contributing to distorted prices and increased market volatility [10–13]. Governmental policies, such as export restrictions, subsidies, and import tariffs, further complicate market predictions, adding layers of unpredictability [14]. Recent challenges, like the COVID-19 pandemic and ongoing geopolitical tensions, have disrupted supply chains, exacerbating the volatility of agricultural markets [15,16].

The nonlinear and interconnected nature of agricultural commodity price fluctuations poses a significant challenge for accurate forecasting, especially amid the growing volatility in global financial and commodity markets. Addressing these challenges requires advanced forecasting models that can capture non-linear patterns and temporal dependencies, which are essential for improving risk management and supporting market stability.

The main contributions and innovations of this study can be summarized in a more concise and academically improved manner as follows:

(a) This study pioneers a hybrid forecasting system for agricultural commodity prices, combining an intelligent optimization algorithm with a deep learning model stack. The proposed SVMD-MSDBO-CNN-BiLSTM-A integrates data decomposition, feature selection, parameter optimization, and forecasting, outperforming nine benchmark models across two datasets.

(b) MSDBO is utilized to optimize six significant hyperparameters of the BiLSTM model, including learning rate, time step, neuron count in hidden layers, training batch size, maximum iterations, and nodes in the fully connected layer. Experimental results indicate a significant improvement in BiLSTM’s forecasting effectiveness.

(c) This study presents MSDBO, which incorporates modified circular chaotic mapping, rat swarm optimization (RSO), golden sine optimization, and an adaptive bidirectional Gaussian perturbation strategy. These modifications enhance the algorithm’s global search capabilities and local exploitation efficiency, while also preventing it from getting trapped in local optima.

The remainder of this article is organized as follows. Sect 2 reviews the relevant literature. Sect 3 introduces the proposed forecasting framework. Sect 4 describes data pre-processing and analysis. Sect 5 presents the experimental results and discussion. Finally, Sect 6 concludes the study and suggests future research directions.

## 2 Literature review

### 2.1 Traditional statistical learning, machine learning, and deep learning methods

Current research in agricultural commodity price forecasting has explored a wide range of methods to develop effective prediction models. These include statistical learning methods, machine learning techniques, deep learning approaches, and hybrid decomposition-ensemble models. Traditional statistical models, such as autoregressive conditional heteroskedasticity (ARCH) [17] and autoregressive integrated moving average (ARIMA) along with its variants [18], have been widely used. However, these models often struggle with nonlinear data, lack generalization capability, and are limited in extracting effective information from time series data. On the other hand, machine learning methods like back propagation neural network (BPNN) [19], extreme learning machine (ELM) [20], and support vector machine (SVM) [21] are prevalent in price forecasting. Despite their widespread application, these methods face challenges such as susceptibility to overfitting.

Recent advancements in deep learning, propelled by big data, have significantly advanced forecasting capabilities. Deep learning models, such as recurrent neural network (RNN) [22], long short-term memory (LSTM) [23], gated recurrent unit (GRU) [24], and Bi-directional long short-term memory (BiLSTM) [25], have proven effective for long-term time series forecasting. They are advantageous over traditional machine learning models due to their interconnected neuron structure in hidden layers [26]. However, deep learning models are not without limitations, often challenged by hyperparameter sensitivity and risks of overfitting or settling in local optima.

### 2.2 Hybrid forecasting models: Weight-based and stack-based

Financial time series, including those for agricultural commodities, are typically complex, chaotic, and nonlinear. Directly forecasting raw data often leads to unsatisfactory results, and no single model can comprehensively predict agricultural commodity prices.

To address these challenges, this study introduces a decomposition-reconstruction framework for agricultural price forecasting. It leverages decomposition denoising techniques to simplify the original time series into less complex subsequences, thereby reducing external noise interference. Prior research has explored various decomposition methods, including empirical wavelet transform (EWT) [27], empirical mode decomposition (EMD) [28], ensemble empirical mode decomposition (EEMD) [29], and successive variational mode decomposition (SVMD) [30].

In agricultural price forecasting, hybrid models are generally divided into two categories: weight-based and stack-based models. For instance, [31] employed three denoising methods—singular spectral analysis (SSA), EMD, and variational mode decomposition (VMD)—to reduce noise in original price data. They then used a combination of statistical learning (ARIMA), machine learning (support vector regression - SVR), and deep learning (RNN, GRU, LSTM) models for forecasting agricultural commodity prices. The weights for each model were optimized using artificial bee colony algorithm (ABC). Similarly, [32] applied EWT, SSA, and VMD for data decomposition, followed by individual forecasts using ARIMA, exponential smoothing (ETS), BPNN, and ELM. Particle swarm optimization (PSO) was then used in conjunction with cuckoo search (CS) for assigning weights to these models.

Stack-based models in price forecasting typically use outputs from basic models as features for higher-level models [33]. For instance, [34] utilized convolutional neural networks (CNNs) to extract features from electricity price data, subsequently inputting these features into a BiLSTM for forecasting. However, research on deep learning stack-based models specifically for agricultural commodity prices is limited.

Deep learning models are increasingly recognized for their suitability in forecasting volatile agricultural commodity prices, yet their effectiveness is heavily influenced by hyperparameters. Optimizing these parameters through intelligent algorithms is crucial for enhanced performance [35]. Recent studies have focused on using optimization algorithms for this purpose, such as genetic algorithm (GA) [36], differential evolutionary algorithm (DE) [37], and sparrow search algorithm (SSA) [38]. However, no single algorithm is universally applicable across all fields, as indicated by the ‘no free lunch’ theorem [39]. For example, [40] employed EWT for input feature decomposition and crisscross optimization algorithm (CSO) for LSTM parameter optimization, enhancing the model’s generalization capability. [41] used DE algorithm to optimize LSTM hyperparameters, achieving superior forecasting accuracy. Similarly, [42] developed a hybrid model combining modified ensemble empirical mode decomposition (MEEMD) with LSTM, optimized by improved whale optimization algorithm (IWOA), outperforming 11 benchmark models.

Although these hybrid forecasting frameworks demonstrate promising performance, several critical challenges remain. First, while decomposition techniques such as EMD, EEMD, and VMD have been widely adopted in agricultural price forecasting, they each have specific drawbacks. EMD suffers from mode mixing, and EEMD—while addressing this issue—introduces stochastic noise. VMD improves frequency separation but may face challenges in handling highly nonstationary signals [43]. In contrast, SVMD exhibits superior decomposition capability, particularly in preserving mode integrity, reducing endpoint effects, and effectively isolating meaningful intrinsic components from noisy time series [44]. These advantages make SVMD a more robust and suitable choice for agricultural price data, which are often nonlinear and volatile. Second, deep learning-based hybrid models often involve numerous hyperparameters; however, many existing studies only tune a limited subset, neglecting important factors such as time steps, hidden neuron count, or activation functions, which significantly influence model accuracy and stability [45]. Third, while optimization algorithms like GA, PSO, and DE have been employed to enhance model performance, their standard versions are susceptible to local optima and often inefficient when navigating complex, high-dimensional hyperparameter spaces [46]. These limitations hinder the robustness, efficiency, and generalization of many existing hybrid forecasting systems.

Based on the aforementioned literature reviews, this study introduces an innovative stack-based deep learning system for forecasting agricultural commodity prices, focusing on parameter optimization and feature extraction. The system, named SVMD-MSDBO-CNN-BiLSTM-A, integrates SVMD, MSDBO, CNN, attention mechanism, and BiLSTM networks. SVMD is employed to break down the original commodity price data into simpler subsequences. CNN and attention mechanism work in tandem to extract relevant information and features from the time series data. MSDBO is merged with BiLSTM to create MSDBO-BiLSTM model, designed for precise forecasting of agricultural commodity prices. MSDBO fine-tunes several parameters of BiLSTM model, including learning rate, number of neurons in the hidden layer, nodes in the fully connected layer, time steps, training batch size, and maximum training iterations. Table 1 lists the abbreviations and their corresponding full names of all models used in this study, and Table 2 subsequently presents a comparative analysis of their advantages and disadvantages.

**Table 1:** List of abbreviations and their full names used in the study.

Abbreviation	Full Name
ARCH	Autoregressive Conditional Heteroskedasticity
ARIMA	Autoregressive Integrated Moving Average
ELM	Extreme Learning Machine
BPNN	Backpropagation Neural Network
LSTM	Long Short-Term Memory
BiLSTM	Bidirectional Long Short-Term Memory
CNN	Convolutional Neural Network
EMD	Empirical Mode Decomposition
EEMD	Ensemble Empirical Mode Decomposition
EWT	Empirical Wavelet Transform
VMD	Variational Mode Decomposition
SVMD	Successive Variational Mode Decomposition
DBO	Dung Beetle Optimization
MSDBO	Multi-Strategy Dung Beetle Optimization
SVMD-BPNN	Successive Variational Mode Decomposition
	– Backpropagation Neural Network
SVMD-LSTM	Successive Variational Mode Decomposition
	– Long Short-Term Memory
SVMD-BiLSTM	Successive Variational Mode Decomposition
	– Bidirectional Long Short-Term Memory
SVMD-CNN-BiLSTM	Successive Variational Mode Decomposition
	– Convolutional Neural Network
	– Bidirectional Long Short-Term Memory
SVMD-CNN-BiLSTM-A	Successive Variational Mode Decomposition
	– Convolutional Neural Network
	– Bidirectional Long Short-Term Memory
	– Attention
VMD-MSDBO-CNN-BiLSTM-A	Variational Mode Decomposition
	– Multi-Strategy Dung Beetle Optimization
	– Convolutional Neural Network
	– Bidirectional Long Short-Term Memory
	– Attention
SVMD-MSDBO-CNN-BiLSTM-A	Successive Variational Mode Decomposition
	– Multi-Strategy Dung Beetle Optimization
	– Convolutional Neural Network
	– Bidirectional Long Short-Term Memory
	– Attention

**Table 2:** Advantages and disadvantages of different forecasting models.

Category	Model	Advantages	Disadvantages	Related work
Physical models	ARCH ARIMA	Easy to build and train models.	Requires large amounts of data and have no capacity to deal with linear features in the data.	[17] [18]
Machine learning methods	BPNN ELM SVM	Have the ability to capture inflection points and have a better forecasting ability.	Have many parameters and can easily become trapped by local optima or overfitting.	[19] [20] [21]
deep learning methods	RNN LSTM GRU BiLSTM	Have the ability to deal with non-linear datasets and capture. long term features.	Inability to richly extract features present in large data.	[22] [23] [24] [25]
Weight-based models	SSA, EMD, VMD- ARIMA, SVR, RNN, GRU and LSTM	Combining the advantages of various models.	Consuming a lot of computing time.	[31]
Stack-based models	CNN-BiLSTM	Can capture abundant features from large-scale data.	Cannot determine hyperparameters.	[34]
Hybrid decomposition-reconstruction models	EWT-CSO-LSTM DE-LSTM MEEMD-IWOA-LSTM	Can capture abundant features from large-scale data and reduce overfitting.	Be difficult to deal with the long-term dependencies of time series.	[40] [41] [42]

## 3 Forecasting model

### 3.1 Framework of developed combined forecasting model

**Fig 1:**
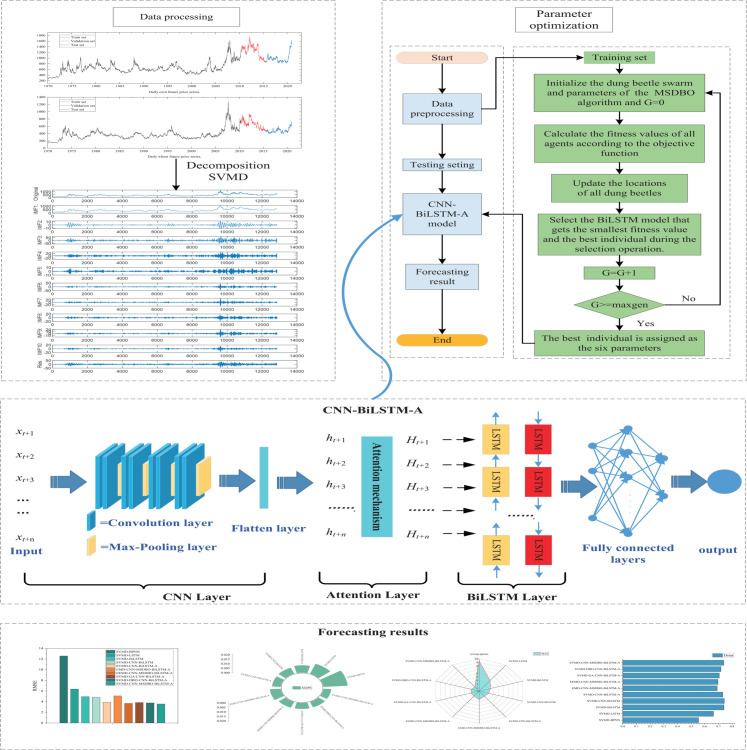
Flowchart of the SVMD-CNN-BiLSTM-A model.

The proposed price forecasting model comprises four main steps, as illustrated in Fig 1.

Step 1: Data collection.

Collect daily future price data for corn and wheat to use as research samples.

Step 2: Data decomposition.

Preprocess the dataset obtained in Step 1. Employ SVMD to decompose the original dataset into several components and divide it into training, validation and test sets.

Step 3: Train MSDBO-CNN-BiLSTM-A models.

This involves initializing the population using improved circular chaos mapping, adopting RSO algorithms for behavioral simulation, and integrating GSA for enhanced optimization. The adaptive bidirectional Gaussian perturbation strategy is applied to avoid local optima. Utilize the training and validation sets from Step 2 to determine the optimal model parameters.

Step 4: Forecasting.

Input the test set data into the trained MSDBO-CNN-BiLSTM-A model to generate the final price forecasting values.

### 3.2 SVMD

SVMD [30] extends the Variational Mode Decomposition (VMD) method by adaptively extracting intrinsic mode functions without predefining their number. Instead of decomposing a signal into a fixed number of modes as in VMD, SVMD successively extracts modes by introducing additional constraints, eliminating the need to specify the number of modes a priori.

Assume the input signal *f*(*t*) can be decomposed into *L*–th order modes *u*_*L*_(*t*) and the residual signal *f*_*r*_(*t*).

$$f(t)=u_{L}(t)+f_{r}(t)$$ (1)

where *u*_*L*_(*t*) is the *L*–th order mode; *f*_*r*_(*t*) is the residual signal, containing the obtained mode $\sum_{i=1:L-1}u_{i}(t)$ and the unprocessed part *f*_*u*_(*t*), which can be expressed as follows:

$$f_{r}(𝔱)=\sum_{i=1:L-1}u_{i}(𝔱)+f_{u}(𝔱)$$ (2)

where *u*_*i*_(*t*) is the *i*-th order mode and the 1st order mode is found by making *f*_*r*_(*t*) in $\left(\Sigma_{i=1:L-1}u_{i}(𝔱)\right)$ be 0. When decomposing the signal to steer the modes predominantly toward the center frequency, the relevant criterion is used as the guiding constraints [47], as shown in the following equation:

$$j_{1}={\left\|\partial_{t}\left[\left(\delta (t)+\frac{j}{\pi t}\right)⊗u_{L}(t)\right]e^{-jw_{L}t}\right\|}_{2}^{2}$$ (3)

where $\omega_{L}$ is the center frequency of the *L*-th mode and ⊗ denotes convolution. To minimize spectral overlap between the residual and the current mode, a correlation criterion is introduced:

$$j_{2}={\left\|\beta_{L}(t)⊗y_{r}(t)\right\|}_{2}^{2}$$ (4)

where $\beta_{L}(t)$ is the impulse response of the filter, its frequency response can be expressed as follows:

$${\hat{\beta }}_{L}(t)=\frac{1}{\alpha (w-w_{L}{)}^{2}}$$ (5)

By minimizing the *j*_1_ and *j*_2_ constraints, the *L* th order modes may not be effectively distinguished from the first *L*-1 order modes. Therefore, based on the idea of establishing constraint *j*_2_, the frequency response of the used filter shown as follows:

$${\hat{\beta }}_{i}(\omega )=\frac{1}{\alpha (\omega -\omega_{i}{)}^{2}},i=1,2,\cdots ,L-1$$ (6)

Thus, the constraints established are expressed as follows:

$$j_{3}=\sum_{i=1}^{L-1}{\left\|\beta_{i}(t)⊗u_{L}(t)\right\|}_{2}^{2}$$ (7)

When performing the decomposition, the following constraints are established to ensure the signal’s complete reconstruct:

$$f(t)=u_{L}(t)+f_{u}(t)+\sum_{i=1:L-1}u_{i}(t)$$ (8)

Therefore, the task of extracting the modal components can be expressed as a constrained minimization problem:

$$\left\{ \begin{array}{l} \min_{u_{L},\omega_{L},f_{r}}\{\alpha j_{1}+j_{2}+j_{3}\}\\ s.t.u_{L}(t)+f_{r}(t)=f(t) \end{array}\right.$$ (9)

where *α* is the parameter for balancing *j*_1_, *j*_2_ and *j*_3_.

### 3.3 CNN

**Fig 2:**
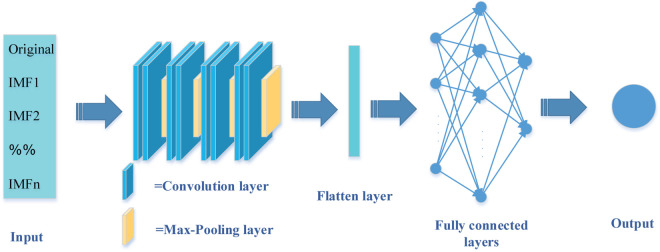
Structure of CNN.

CNNs, proposed by LeCun *et al*. [48], are advanced variants of traditional back propagation networks. They are classified by the dimension of convolution: 1D-CNNs for sequences, 2D-CNNs for images, and 3D-CNNs for videos and volumetric data. Key principles of CNNs include local receptive fields, weight sharing, and pooling, which collectively reduce the number of parameters and enhance computational efficiency. A typical CNN consists of three components: the convolutional layer, the pooling layer, and the fully connected layer. The convolutional layer extracts features through convolution operations, while the pooling layer downsamples these features to reduce dimensionality and computational cost. Fig 2 illustrates the structure of a CNN.

$$p_{t}=\tanh(x_{t}*w_{t}+b_{t})$$ (10)

Where *p*_*t*_ is the the output value after convolution; tanh is the activation function; *x*_*t*_ is the input vector; *w*_*t*_ is the weight of the convolution kernel; and *b*_*t*_ is the bias of the convolution kernel.

### 3.4 Attention mechanism

The attention mechanism, inspired by the human brain’s focus system, operates on a principle of probability weighting [49]. Its core concept is to prioritize important information by assigning it higher weights, while less significant information receives lower weights. In the context of forecasting price series, the attention mechanism is incorporated to improve the feature extraction capabilities of CNNs when analyzing decomposed subsequences and residuals. The formula for the attention mechanism is outlined as follows:

$$e_{i}=\tanh(wh_{i}+b)$$ (11)

$$w_{i}=\frac{\exp(e_{i})}{\sum_{j=1}^{t}\exp(e_{j})}$$ (12)

$$y=\sum_{i=1}^{t}w_{i}h_{i}$$ (13)

where: *h*_*i*_ is the hidden state at time step *i* from the input sequence. *W* and *b* are the learnable weight matrix and bias vector of the scoring network, respectively. *e*_*i*_ is the unnormalized attention score for *h*_*i*_. $\alpha_{i}$ (or *w*_*i*_) is the normalized attention weight assigned to *h*_*i*_. *t* is the total length of the input sequence. *y* is the context vector, a weighted representation of the most relevant parts of the input sequence.

### 3.5 BiLSTM

**Fig 3:**
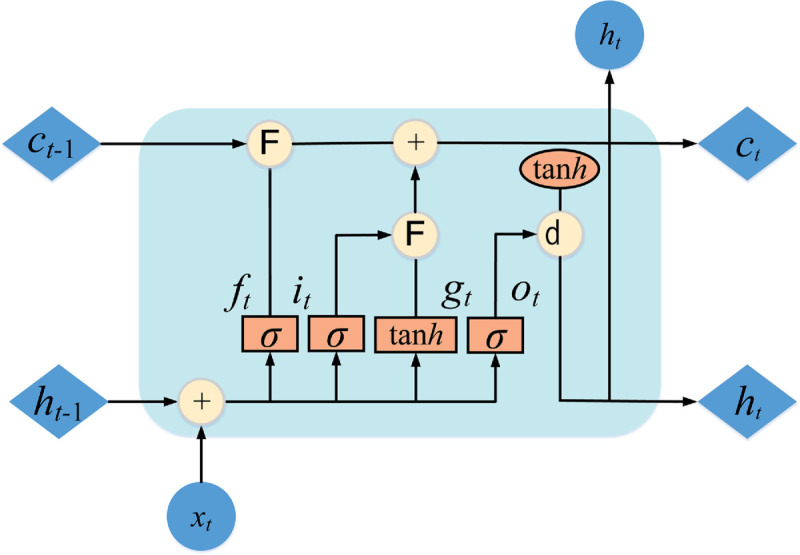
One basic unit of LSTM.

LSTM, a variant of RNN, was developed by Hochreiter & Schmidhuber to overcome the issue of long-term dependencies that RNN models face [50]. A BiLSTM consists of two LSTM layers, one processing data forwards and the other backwards, hence the term ‘bidirectional’. The architecture of LSTM includes four main components: a forget gate, an input gate, an output gate, and a cell state. The structure of a single-layer LSTM is illustrated in Fig 3.

The sigmoid function is defined as:

$$\sigma (x)=\frac{1}{1+e^{-x}}$$ (14)

The forgetting gate is expressed as follows:

$$f(t)=\sigma \left(w_{f}\;*\;\left[h_{t-1},{\tilde{X}}_{t}\right]+b_{f}\right)$$ (15)

The inputting gate is defined as follows:

$$i_{t}=\sigma \left(w_{i}\;*\;\left[h_{t-1},{\tilde{X}}_{t}\right]+b_{i}\right)$$ (16)

The cell state update is a two-step process. First, a candidate cell state *g*_*t*_ is created using a tanh layer:

$$g_{t}=\tan\;h\left(W_{c}*\left[h_{\left(t-1\right)},{\tilde{X}}_{t}\right]+b_{c}\right)$$ (17)

The state value of the memory cell is written as follows:

$$C_{t}=f_{t}\;*\;C_{t-1}+i_{t}*{\tilde{C}}_{t}$$ (18)

The output of the output gate is expressed as follows:

$$o_{t}=\sigma (w_{o}\;*\;[h_{t-1},\tilde{X_{t}}]+b_{o})$$ (19)

The output of the hidden layer is defined as follows:

$$h_{t}=o_{t}*\tan\;h(C_{t})$$ (20)

The backward LSTM calculation in the BiLSTM structure is similar to the forward LSTM, and the BiLSTM is determined as follows.

$$h_{f}=f\left(w_{f_{1}}\;*\;{\tilde{X}}_{t}+w_{f2}\;*\;h_{t-1}\right)$$ (21)

$$h_{b}=f\left(w_{b_{1}}\;*\;{\tilde{X}}_{t}+w_{b2}\;*\;h_{t+1}\right)$$ (22)

where *h*_*f*_ is the output of the forward LSTM network and *h*_*b*_ is the output of the reverse LSTM network. The final output of the hidden layer is shown as follows:

$$y_{t}=\sigma \left(w_{yh}\;*\;h_{f}+b_{y}\right)$$ (23)

where, *σ* is the sigmoid activation function; tan h is the hyperbolic tangent activation function; the matrices *W*_*f*_, *W*_*i*_, *W*_*c*_, *W*_*o*_, *w*_*f*1_, *w*_*f*2_, *w*_*b*1_, *w*_*b*2_ are the recurrent weight matrices; *w*_*hy*_ is the hidden output weight matrix; *b*_*f*_, *b*_*i*_, *b*_*c*_, *b*_*o*_, *b*_*y*_ are the bias vectors; and *y*_*t*_ is the final hidden layer output.

**Fig 4:**
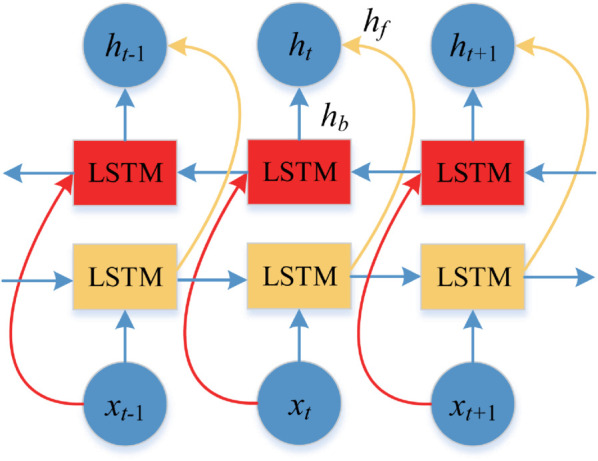
Structure of BiLSTM network.

Traditional neural networks often fail to fully utilize time series data for agricultural commodity prices, resulting in suboptimal forecasting performance. In contrast, BiLSTM, with its bi-directional architecture, improves forecasting accuracy by using two LSTM layers working in opposite directions to leverage both past and future information. This helps address data insufficiency issues commonly seen in standard LSTM models [51]. The structural design of BiLSTM model is depicted in Fig 4.

### 3.6 Improved dung beetle optimizer

The Dung Beetle Optimizer (DBO) is a swarm intelligence algorithm inspired by dung beetles’ behaviors such as rolling, dancing, stealing, foraging, and reproduction [52]. While the original DBO exhibits promising global search ability, it suffers from slow convergence and imbalanced exploration–exploitation. To address these limitations, we propose a an improved MSDBO with the following enhancements:

(a) Improved circle chaotic mapping.

Chaotic mapping is known for its thoroughness and randomness in exploring solutions. Utilizing chaotic mapping functions to generate initial positions for population individuals in an algorithm can improve the uniformity and coverage of the initial solution space. This technique has been successfully applied in areas such as artificial neural networks, modeling of natural phenomena, and nonlinear circuits, demonstrating significant effectiveness [53]. Common types of chaos maps include logistic, tent, and circle chaos maps. Among these, circular chaotic mapping is particularly notable for its stability and comprehensive coverage of chaotic values [54]. The formula for the improved circular chaotic mapping is detailed in Eq (24).

$$x_{i+1}=mod\left(x_{i}+\alpha -\left(\frac{\beta }{2\pi }\right)\sin\left(2\pi x_{i}\right),1\right)$$ (24)

Where, *α* = 0.4204, *β*=0.0305.

(b) Fusion RSO and DBO.

DBO has been augmented by incorporating rat swarm optimization (RSO) algorithm, specifically to enhance the ball-rolling behavior of dung beetles. RSO algorithm, inspired by the chasing and fighting behaviors observed in rat swarms, is a recent development in group intelligence algorithms [55]. Its simplicity and straightforward structure, devoid of complex mathematical operations, make it effective in both global searching and local exploitation. By adopting RSO’s chasing and fighting strategies in place of the dung beetle’s traditional ball-rolling and dancing behaviors, the optimization process is further improved. The prey-chasing behavior integral to RSO is concisely represented in Eqs (25)–(27).

$$P=A\times x_{i}(t)+C\times (x_{b}(t)-x_{i}(t))$$ (25)

$$A=a-t\times \frac{a}{T_{\max}}$$ (26)

C=2×rand() (27)

Where: *x*_*b*_(*t*) is the current optimal position of the rat population; *x*_*i*_(*t*) is the current optimal position of the rat population; *x*_*i*_ a is the random number in [1, 5]; rand is the random number in [0, 1]; *t* is the current iteration number; and *T*_*m*_*ax* is the maximum number of iterations.

In the modeling of RSO, the fight process between the rats and prey is defined in Eq (28).

$$x_{i}(t+1)=|x_{b}(t)-P|$$ (28)

Where: *x*_*i*_(*t* + 1) is the location of the (t+1)–th rat population.

(c) Fusion of GSA and DBO.

The optimization capabilities of DBO have been enhanced through the integration of golden sine algorithm (GSA), a novel metaheuristic proposed by Tanyildizi and Demir in 2017 [56]. This algorithm uses a sine function to model optimization problems. By incorporating the golden section coefficient, GSA effectively searches around potential solutions, enhancing the local exploitation capability and accelerating the convergence process of the overall algorithm. This method replaces the stealing behavior typically observed in dung beetles. The adaptation of this modified local search strategy within the algorithm is outlined in Eq (29).

$$x_{i}^{t+1}=x_{i}^{t}*|\sin(R_{1})|-R_{2}*\sin(R_{1})*|x_{1}*P_{i}^{t}-x_{2}*x_{i}^{t}|$$ (29)

Where, *R*_1_ and *R*_2_ are random numbers between [0, 2*π* ] and [0, *π*], respectively. *R*_1_ denotes the distance moved, and *R*_2_ denotes the direction of movement; $P_{i}^{t}$ denotes the position of the *i*-th dung beetle optimal individual at the *t*-th iteration; *x*_1_, and *x*_2_ are the coefficients obtained by introducing the number of golden divisors, which can reduce the search space and make the individual gradually close to the optimal value, these parameters can be expressed as *x*_1_, *x*_2_ $x_{1}=-\pi +(1-\tau )*2\pi$, $x_{2}=-\pi +\tau *2\pi$; and *τ* is the golden section number, with the utilization of irrational number 3.

(d) Adaptive bidirectional gaussian perturbation strategy.

To prevent the algorithm from getting trapped in local optima, an adaptive bidirectional gaussian perturbation strategy is employed [57]. This strategy updates the positions of the current individuals in the algorithm, allowing it to effectively escape from local optimal solutions. The mechanism of this perturbation is detailed in Eq (30). Additionally, Gaussian perturbation factor U is defined in Eq (31).

$$\left\{ \begin{array}{cc} x_{i}(t+1)=x_{i}(t)+U*((U_{b}-L_{b})*r+L_{b}) & r\geq 0.5\\ x_{i}(t+1)=x_{i}(t)-U*((U_{b}-L_{b})*r+L_{b}) & r<0.5 \end{array}\right.$$ (30)

$$U=1-\sqrt{t/T_{\max}}$$ (31)

### 3.7 Pseudocode of the proposed MSDBO

To improve accessibility, the overall procedure of the proposed MSDBO is summarised in Algorithm 1. The four core behaviours—(i) **Ball rolling (RSO-enhanced)**, (ii) **Breeding**, (iii) **Foraging**, and (iv) **Stealing (GSA-enhanced)**—are executed iteratively. Optional modules such as improved circle chaotic initialization and adaptive bi-directional Gaussian perturbation are inserted at the corresponding stages.

**Algorithm 1. Improved Multi-Strategy Dung Beetle Optimizer (MSDBO).**



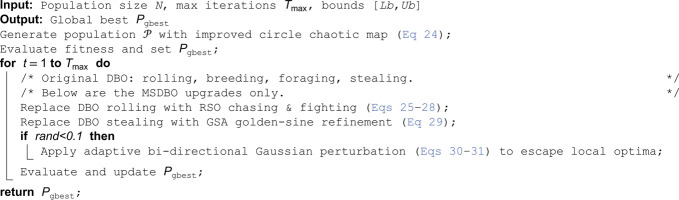



### 3.8 Convergence evaluation of MSDBO

**Fig 5:**
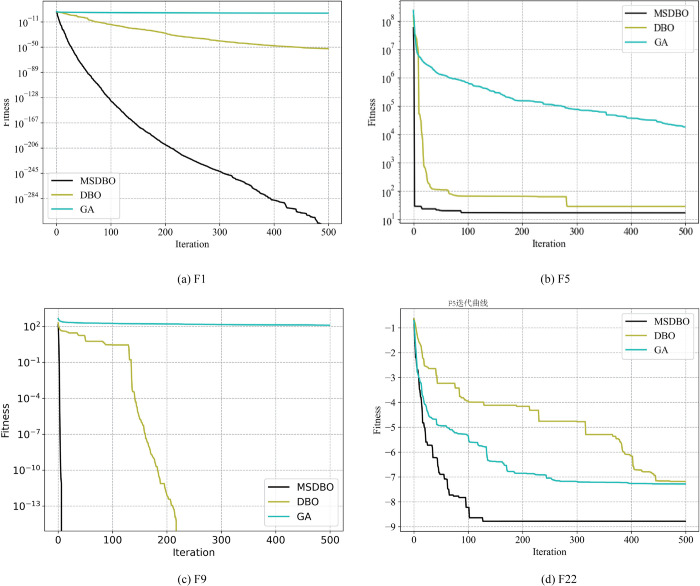
Convergence curves for two unimodal benchmark functions and two multimodal functions.

Four benchmark functions, commonly used for evaluating algorithm performance, were employed in this work. These functions were selected based on the study by GhaemiDizaji *et al*.[58]. The convergence behavior of the proposed multi-strategy enhanced dung beetle optimizer (MSDBO) on these benchmark functions is illustrated in Fig 5. Among them, F1 and F5 are unimodal functions, shown in Fig 5(a) and Fig 5(b), respectively, while F9 and F22 are multimodal functions, presented in Fig 5(c) and Fig 5(d).

For the unimodal functions F1 and F5, MSDBO demonstrates a significantly faster convergence rate during the initial iterations, rapidly approaching the optimal solution. This performance clearly surpasses that of the original DBO and the GA, indicating the superior global exploration and local exploitation capabilities of MSDBO. In the case of the multimodal functions F9 and F22, MSDBO continues to exhibit robust optimization behavior, effectively escaping local optima and maintaining stable convergence. These results further confirm the algorithm’s strong local search ability and robustness in avoiding premature convergence.

## 4 Experimental

### 4.1 Data description

**Fig 6:**
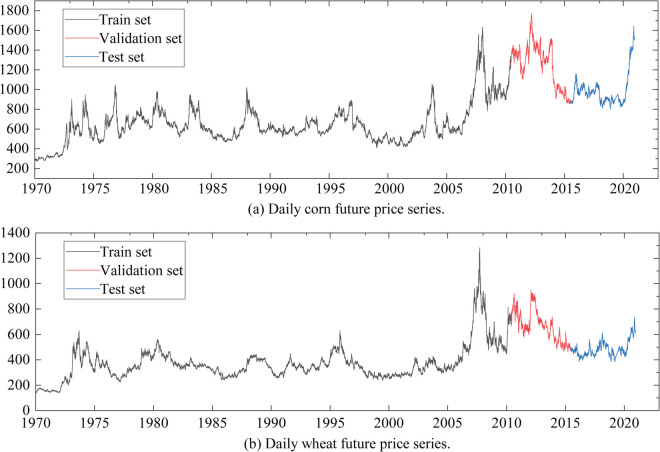
Daily corn and wheat future price series.

The Chicago Board of Trade (CBOT) provides globally recognized benchmark prices for bulk agricultural commodities, reflecting market supply-demand dynamics, macroeconomic shocks, and investor sentiment. These price data are essential for analyzing nonlinear price movements and capturing high-frequency volatility, offering a robust foundation for understanding price dynamics, improving risk assessment, and supporting financial market stability [31]. In this context, this study utilizes daily closing price data of corn and wheat traded on the CBOT to conduct an in-depth analysis . The dataset spans from January 1970 to May 2021, comprising 12,850 price observations for corn and 12,856 for wheat. The data is segmented into three parts: the first 80% is designated as the training set, the subsequent 10% as the validation set, and the final 10% as the test set. Fig 6(a) and 6(b) show the corn and wheat price datasets, respectively. Table 3 summarises the main statistical information of the data.

**Table 3:** Descriptive statistics for agricultural commodity price prediction.

Dataset	Statistical values						
	Number	Min	Max	Mean	Standard deviation	Skewness	Kurtosis
Corn	12850	112.00	831.25	302.84	126.65	1.62	3.02
Wheat	12856	135.38	1282.50	408.00	156.20	1.22	2.07

### 4.2 Evaluation criteria

The model’s forecasting performance is assessed using four key metrics: root mean square error (RMSE), mean absolute error (MAE), mean absolute percentage error (MAPE), and a directional accuracy *D*_*stat*_.

$$MAE=\frac{1}{N}\sum_{i=1}^{N}|{\hat{y}}_{i}-y_{i}|$$ (32)

$$RMSE=\sqrt{\frac{1}{N}\sum_{i=1}^{N}({\hat{y}}_{i}-y_{i}{)}^{2}}$$ (33)

$$MAPE=\frac{1}{N}\sum_{i=1}^{N}|\frac{{\hat{y}}_{i}-y_{i}}{y_{i}}|\times 100\%$$ (34)

$$D_{stat}=\frac{1}{N}\sum_{t=1}^{N}\theta_{t}$$ (35)

Where *N* is the number of output samples; ${\hat{y}}_{i}$ denotes the forecasting value; *y*_*i*_ denotes the actual value; and $\theta_{t}$=1 only when $(y_{i+1}-y_{i})({\hat{y}}_{i+1}+{\hat{y}}_{i})\geq 0$ and $\theta_{t}$ = 0 otherwise.

### 4.3 Decomposition and denoising

**Fig 7:**
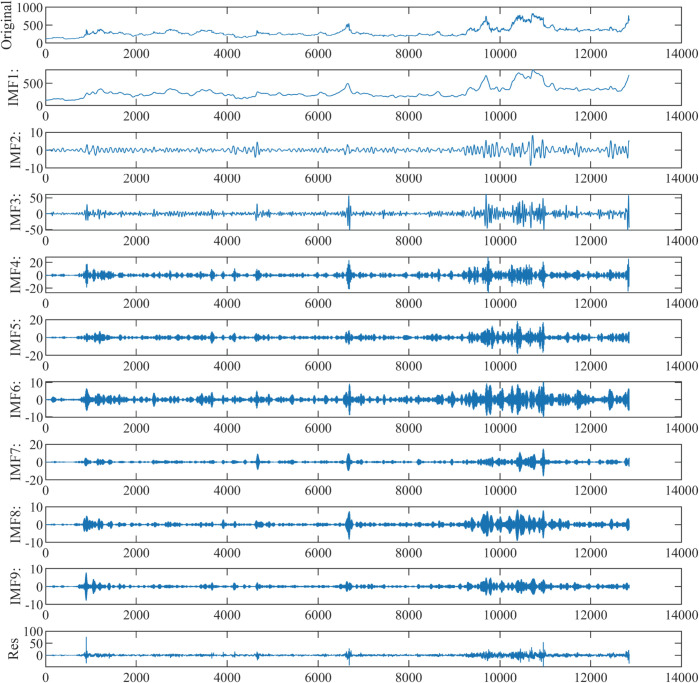
The decomposed components of corn.

**Fig 8:**
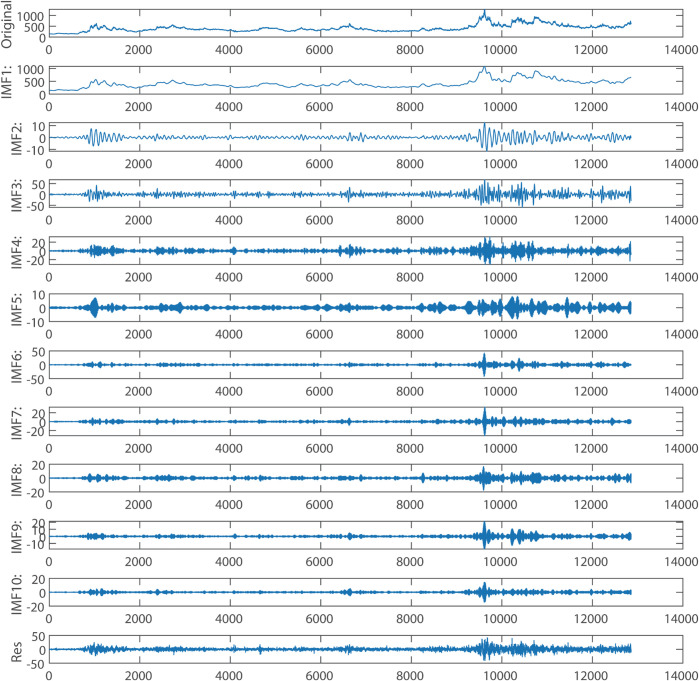
The decomposed components of wheat.

To mitigate the nonlinearity and high volatility in the price data, thereby enhancing prediction accuracy, the original corn data was decomposed using SVMD, resulting in 9 Intrinsic Mode Functions (IMFs) and a residual series. Similarly, the wheat data decomposition yielded 10 IMFs and a residual series. Figs 7 and 8 display the original corn and wheat data along with their respective SVMD decompositions.

## 5 Forecasting analysis

### 5.1 Comparable models and parameter setting

To assess the accuracy and stability of the proposed model, its performance is compared against nine benchmark models, including variants based on SVMD, EMD, and EEMD combined with BPNN, LSTM, BiLSTM, CNN-BiLSTM, and optimization strategies such as DBO and MSDBO.

BPNN, LSTM, and BiLSTM were also included as standalone benchmark models. Their parameters were carefully tuned, with BiLSTM time steps set between 1 and 48, hidden layers set to two layers, up to 200 training epochs, 1–128 hidden neurons, batch sizes from 1 to 120, and learning rates between 0.001 and 0.5. Full parameter settings are listed in Table 4.

**Table 4:** Comparison of model parameter settings.

Comparision Model	Experimental parameters	Corn	Wheat
BiLSTM/LSTM	Hidden layers	2	2
	Hidden neurons	25	26
	Learning rate	0.001	0.001
	Batch size	32	32
	Time steps	24	24
	Epochs	1000	1000
	Optimizer	Adam	Adam
BPNN	Epochs	1000	1000
	Training requirements precision	0.00004	0.00004
	Optimizer	Adam	Adam
	Learning rate	0.001	0.001
	Time steps	24	24

### 5.2 Comparison of the proposed model with other established comparable models

**Fig 9:**
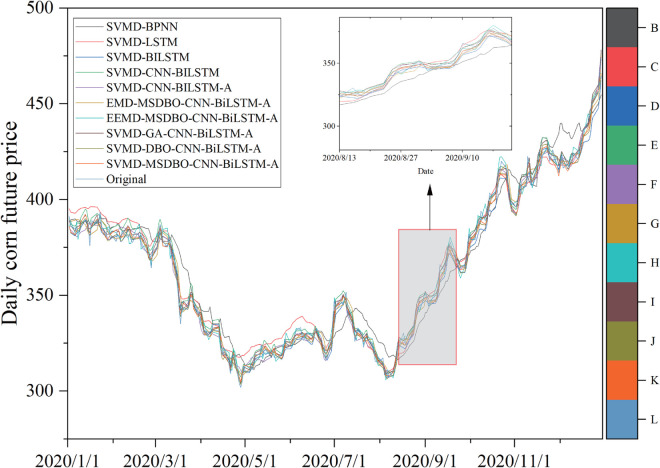
Performance evaluation of forecast combination models for corn.

**Fig 10:**
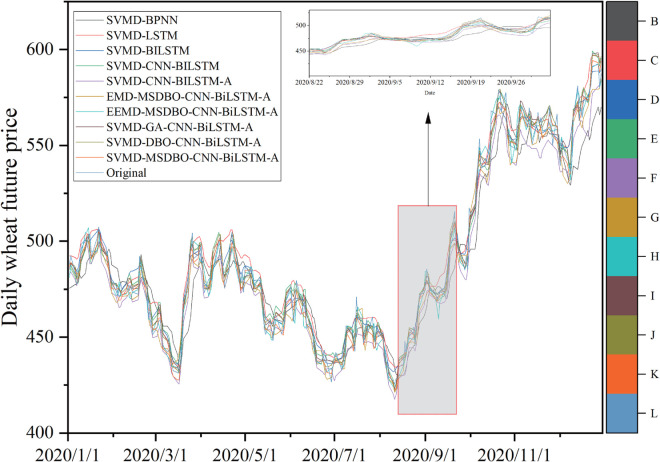
Performance evaluation of forecast combination models for wheat.

**Fig 11:**
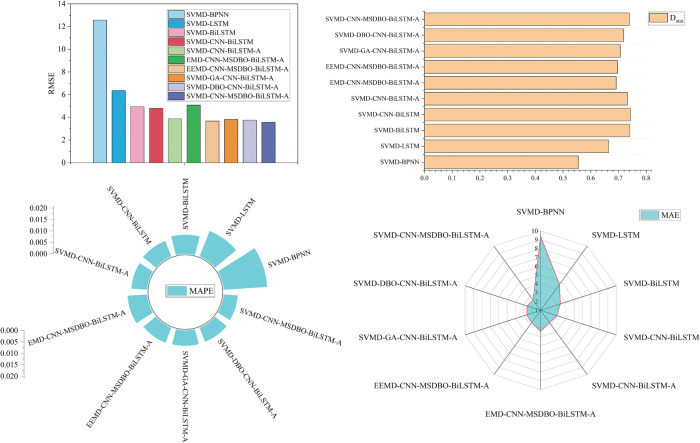
RMSE, MAE, $D_{stat}$, and MAPE of combination models for corn price forecasting.

**Fig 12:**
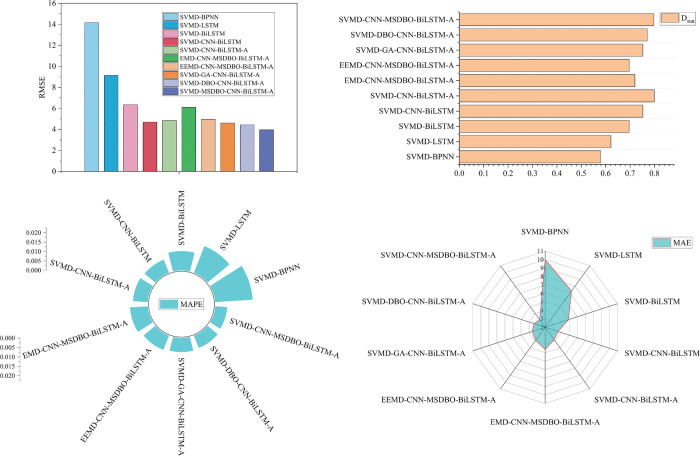
RMSE, MAE, $D_{stat}$, and MAPE of combination models for wheat price forecasting.

The proposed SVMD-BiLSTM model is evaluated using RMSE, MAPE, MAE, and *D*_*stat*_, and compared with nine alternative models. Forecasting results for corn and wheat are presented in Figs 9 and 10, while corresponding error metrics are shown in Figs 11 and 12. Detailed performance comparisons are summarized in Tables 5 and 6, followed by an in-depth discussion.

**Table 5:** Comparative of forecasting performance by different models for corn prices.

Models	Corn			
	RMSE/ $IR_{RMSE}$	MAE/ $IR_{MAE}$	MAPE/ $IR_{MAPE}$	$D_{stat}$ $IR_{D}$
SVMD-BPNN	12.5744	9.4117	1.9051%	55.4864%
	(71.61%)	(73.85%)	(67.62%)	(–33.20%)
SVMD-LSTM	6.3569	4.6458	1.1969%	66.4330%
	(43.84%)	(47.03%)	(48.46%)	(–11.25%)
SVMD-BiLSTM	4.9433	3.3500	0.8312%	73.0654%
	(27.78%)	(26.53%)	(25.78%)	(–1.15%)
SVMD-CNN-BILSTM	4.8075	2.952	0.7267%	74.2991%
	(25.74%)	(16.63%)	(15.11%)	(0.52%)
SVMD-CNN-BiLSTM-A	3.8765	2.7274	0.6866%	73.2087%
	(7.90%)	(9.76%)	(10.15%)	(–0.96%)
SVMD-MSDBO-CNN-BiLSTM-A	**3.5701**	**2.4611**	**0.6169%**	**73.9097%**

**Table 6:** Comparative of forecasting performance by different models for wheat prices.

Models	Wheat			
	RMSE/ $IR_{RMSE}$	MAE/ $IR_{MAE}$	MAPE/ $IR_{MAPE}$	*D*_*stat*_ $IR_{D}$
SVMD-BPNN	14.1507	10.0106	1.9913%	57.9767%
	(71.94%)	(69.02%)	(67.98%)	(–37.58%)
SVMD-LSTM	9.1379	7.2992	1.5157%	62.1790%
	(56.55%)	(57.51%)	(57.93%)	(–28.29%)
SVMD-BiLSTM	6.3368	4.9047	1.0214%	69.6498%
	(37.35%)	(36.76%)	(37.57%)	(–14.53%)
SVMD-CNN-BILSTM	4.8336	3.8085	0.7806%	78.3222%
	(17.86%)	(18.56%)	(18.31%)	(–1.84%)
SVMD-CNN-BiLSTM-A	4.4281	3.4012	0.7010%	77.0428%
	(10.34%)	(8.81%)	(9.03%)	(–3.54%)
SVMD-MSDBO-CNN-BiLSTM-A	**3.9701**	**3.1016**	**0.6377%**	**79.7665%**

(a) SVMD-BiLSTM model shows superior results on both datasets compared to SVMD-BPNN and SVMD-LSTM, achieving the lowest RMSE, MAE, MAPE, and maximum *D*_*stat*_. Specifically, for the corn dataset, the MAPE values for SVMD-BPNN, SVMD-LSTM, and SVMD-BiLSTM are 1.9051%, 1.1969%, and 0.8312%, respectively. These results establish BiLSTM as the most effective individual forecasting model in this comparison.

(b) A comparison between SVMD-CNN-BiLSTM and SVMD-BiLSTM on both datasets shows that the former consistently outperforms the latter across all evaluation metrics. For instance, on the corn dataset, SVMD-CNN-BiLSTM achieves an RMSE of 4.8075, MAE of 2.952, MAPE of 0.7267%, and *D*_*stat*_ of 74.2991%. These results highlight the effectiveness of CNN in extracting features and enhancing forecasting accuracy.

(c) Further improvement is observed with the incorporation of an attention mechanism in SVMD-CNN-BiLSTM-A, which leads to better performance on both datasets. On the wheat dataset, it achieves an RMSE of 3.9701, MAE of 3.1016, MAPE of 0.6377%, and *D*_*stat*_ of 79.7665%, confirming the contribution of the attention mechanism in improving model precision when integrated with CNN.

(d) When comparing SVMD-CNN-BiLSTM-A with SVMD-MSDBO-CNN-BiLSTM-A, the latter yields the best performance on both datasets. On the corn dataset, RMSE, MAE, and MAPE are reduced by 7.90%, 9.76%, and 10.15%, respectively, while *D*_*stat*_ increases by 0.96%. These findings demonstrate that integrating optimization algorithms with deep hybrid models significantly enhances forecasting effectiveness.

### 5.3 Comparison of different decomposition methods

**Fig 13:**
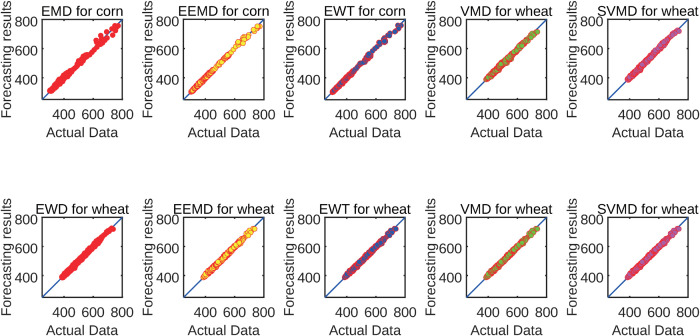
Scatter plots of actual and forecasted values of different decomposition methods for corn and wheat datasets.

This section evaluates the performance of various signal decomposition techniques—including EMD, EEMD, EWT, VMD, and SVMD—within a consistent forecasting framework. Fig 13 illustrates the scatter plot of the forecasting and true values for different decomposition methods based on the same forecasting framework. Tables 7 and 8 demonstrate that SVMD consistently outperforms other decomposition methods in forecasting accuracy for both corn and wheat prices, while Table 9 lists the corresponding p-values from Wilcoxon rank-sum tests, statistically validating these improvements.

**Table 7:** Comparative results of by different decomposition methods for corn prices.

Models	Corn			
	RMSE/ $IR_{RMSE}$	MAE/ $IR_{MAE}$	MAPE/ $IR_{MAPE}$	$D_{stat}$ $IR_{D}$
EMD-MSDBO-CNN-BiLSTM-A	5.0855	3.3752	0.8564%	69.1589%
	(29.80%)	(27.08%)	(27.97%)	(–6.87%)
EEMD-MSDBO-CNN-BiLSTM-A	3.6759	2.7218	0.7029%	69.7040%
	(2.88%)	(9.58%)	(12.24%)	(–6.03%)
EWT-MSDBO-CNN-BiLSTM-A	3.7047	2.6356	0.6636%	71.1838%
	(3.77%)	(7.07%)	(7.94%)	(–3.68%)
VMD-MSDBO-CNN-BiLSTM-A	3.5845	2.4709	0.6227%	71.8847%
	(0.40%)	(0.40%)	(0.93%)	(–2.75%)
SVMD-MSDBO-CNN-BiLSTM-A	**3.5701**	**2.4611**	**0.6169%**	**73.9097%**

**Table 8:** Comparative results of by different decomposition methods for wheat prices.

Models	Wheat			
	RMSE/ $IR_{RMSE}$	MAE/ $IR_{MAE}$	MAPE/ $IR_{MAPE}$	$D_{stat}$ $IR_{D}$
EMD-MSDBO-CNN-BiLSTM-A	6.0983	4.6285	0.9412%	71.9844%
	(34.90%)	(32.99%)	(32.25%)	(–10.81%)
EEMD-MSDBO-CNN-BiLSTM-A	4.9611	3.9127	0.8113%	69.6498%
	(19.98%)	(20.73%)	(21.40%)	(–14.53%)
EWT-MSDBO-CNN-BiLSTM-A	4.5195	3.4639	0.7062%	74.9416%
	(13.27%)	(13.88%)	(9.43%)	(–4.76%)
VMD-MSDBO-CNN-BiLSTM-A	4.4281	3.3958	0.6986%	75.1751%
	(11.57%)	(11.31%)	(3.07%)	(–3.20%)
SVMD-MSDBO-CNN-BiLSTM-A	**3.9701**	**3.1016**	**0.6377%**	**79.7665%**

(b) Similarly, for the wheat dataset, SVMD again delivers the best performance across all metrics, with RMSE of 3.9701, MAE of 3.1016, MAPE of 0.6377%, and *D*_*stat*_ of 79.7665%. Compared with the EEMD-based model, SVMD improves RMSE by 19.98%, MAE by 20.73%, MAPE by 21.40%, and *D*_*stat*_ by 14.53%. When compared to the newly included EWT and VMD models, SVMD still offers noticeable advantages, further validating its effectiveness.

(c) These findings highlight that the SVMD-based decomposition approach provides the most significant improvements in forecasting performance. Its superior ability to extract relevant features contributes to lower prediction errors and better directional accuracy, making it highly effective for agricultural futures forecasting tasks.

(d) The statistical significance of performance differences was rigorously examined using the Wilcoxon rank-sum test, with all *p*-values documented in Table 9. A *p*-value exceeding the 0.05 threshold indicates no statistically significant difference between decomposition methods, whereas values below 0.05 confirm significant performance disparities. Our analysis demonstrates SVMD’s decisive superiority in agricultural markets: extreme significance over EMD (corn: *p* = 3.17 × 10^−5^<0.05; wheat: $p=2.45\times 10^{-6}<0.05$) and consistent advantages against VMD (corn: *p* = 0.0147<0.05; wheat: $p=1.89\times 10^{-4}<0.05$). These systematically robust outcomes validate SVMD’s dual strengths in agricultural price forecasting - numerical dominance and statistical rigor.

**Table 9:** Comparison of P-values between SVMD-MSDBO-CNN-BiLSTM-A and other methods.

Models	Corn	Wheat
EMD-MSDBO-CNN-BiLSTM-A	$3.17\times 10^{-5}$	$2.45\times 10^{-6}$
EEMD-MSDBO-CNN-BiLSTM-A	$1.92\times 10^{-3}$	$5.14\times 10^{-5}$
EWT-MSDBO-CNN-BiLSTM-A	$9.76\times 10^{-4}$	$3.02\times 10^{-6}$
VMD-MSDBO-CNN-BiLSTM-A	$1.47\times 10^{-2}$	$1.89\times 10^{-4}$
SVMD-MSDBO-CNN-BiLSTM-A	$3.5701\times 10^{-5}$	$3.1016\times 10^{-6}$

### 5.4 Comparison of different optimization algorithm

**Fig 14:**
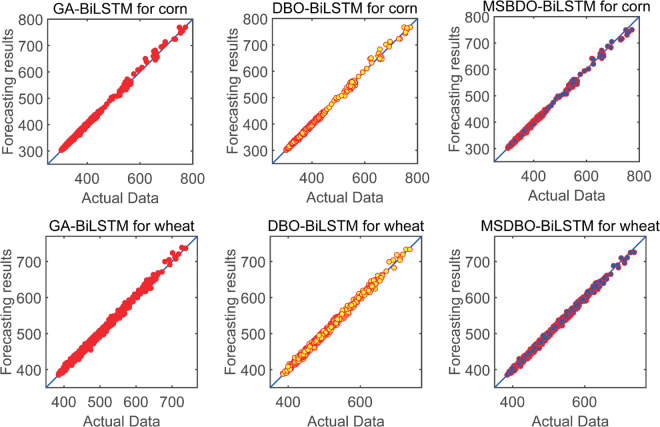
Scatter plots of actual and forecasted values of different optimization algorithms for BiLSTM models in corn and wheat datasets.

This section analyzes the impact of optimization method selection on forecasting accuracy, with particular focus on demonstrating the enhanced performance of the improved DBO algorithm. Fig 14 illustrates the scatter plot of the forecasting and true values for different optimization methods based on the same forecasting framework. Tables 10 and 11 show the forecasting results of the forecasting model framework based on three different optimization methods for two different datasets. By analyzing the results in Tables 10 and 11, the analysis can be summarized as follows.

**Table 10:** Comparison of forecasting performance by different models for corn prices.

Models	Corn			
	RMSE/ $IR_{RMSE}$	MAE/ $IR_{MAE}$	MAPE/ $IR_{MAPE}$	$D_{stat}$ $IR_{D}$
SVMD-GA-CNN-BiLSTM-A	3.8298	2.6704	0.6755%	70.6386%
	(6.78%)	(7.84%)	(8.68%)	(–4.63%)
SVMD-DBO-CNN-BiLSTM-A	3.7602	2.6104	0.6566%	71.8847%
	(5.06%)	(5.72%)	(6.05%)	(–2.82%)
SVMD-MSDBO-CNN-BiLSTM-A	**3.5701**	**2.4611**	**0.6169%**	**73.9097%**

**Table 11:** Comparison of forecasting performance by different models for wheat prices.

Models	Wheat			
	RMSE/ $IR_{RMSE}$	MAE/ $IR_{MAE}$	MAPE/ $IR_{MAPE}$	$D_{stat}$ $IR_{D}$
SVMD-GA-CNN-BiLSTM-A	4.6047	3.6089	0.7431%	75.2529%
	(13.78%)	(14.06%)	(14.18%)	(–6.00%)
SVMD-DBO-CNN-BiLSTM-A	4.4281	3.4012	0.7010%	77.0428%
	(10.34%)	(8.81%)	(9.03%)	(–3.54%)
SVMD-MSDBO-CNN-BiLSTM-A	**3.9701**	**3.1016**	**0.6377%**	**79.7665%**

(a) Tables 10 and 11 demonstrate that the MSDBO based forecasting framework achieves minimum values for RMSE, MAE and MAPE and maximum values for *D*_*stat*_ on both data. For example, on the corn dataset, for MAPE, the MSDBO based forecasting framework obtains an reduction of 8.68% and 6.05% compared to the GA and DBO based forecasting frameworks, respectively. These results suggest that MSDBO can better optimize the model parameters and improve the forecasting accuracy of the model.

(b) Further examination of Tables 10 and 11 demonstrates that MSDBO-based framework outperforms DBO-based framework. This means that four strategies can be used to improve DBO. For example, within the wheat dataset, the application of MSDBO results in a 10.34% reduction in RMSE, an 8.81% reduction in MAE, a 9.03% reduction in MAPE, and a 3.54% improvement in *D*_*stat*_. Besides that, the efficacy of optimization algorithms combined with stack-based models in price forecasting is further demonstrated.

### 5.5 Comparison of the forecasting speed

In this section, we analyze the forecasting speed of different models under a unified CNN-BiLSTM architecture. The experimental results are shown in Table 12. The main findings can be summarized as follows:

**Table 12:** Comparison of prediction efficiency by different models.

Models	Corn	Wheat
	Run time (s) $IR_{Runtime}$	Run time (s) $IR_{Runtime}$
SVMD-GA-CNN-BiLSTM-A	553.1687	582.0364
	(26.72%)	(26.66%)
SVMD-DBO-CNN-BiLSTM-A	473.4415	502.5751
	(14.40%)	(15.06%)
EMD-MSDBO-CNN-BiLSTM-A	425.3236	441.1789
	(4.70%)	(3.33%)
EEMD-MSDBO-CNN-BiLSTM-A	446.3782	463.7592
	(–10.12%)	(–8.66%)
EWT-MSDBO-CNN-BiLSTM-A	402.8574	411.2543
	(0.61%)	(3.65%)
VMD-MSDBO-CNN-BiLSTM-A	425.2588	443.5782
	(4.68%)	(3.76%)
SVMD-MSDBO-BiLSTM	305.1220	314.5482
	(–24.72%)	(–26.31%)
SVMD-MSDBO-CNN-BiLSTM	344.2571	362.0145
	(–15.07%)	(–15.19%)
**SVMD-MSDBO-CNN-BiLSTM-A**	**405.3345**	**426.8496**

(a) The SVMD-MSDBO-CNN-BiLSTM-A model is selected as the performance benchmark, achieving execution times of 405.33 seconds and 426.85 seconds on the Corn and Wheat datasets, respectively. Despite incorporating multilevel signal decomposition and metaheuristic optimization, its overall runtime remains within an acceptable range, demonstrating good practical feasibility.

(b) Compared to traditional signal decomposition methods, the SVMD-MSDBO-CNN-BiLSTM-A model exhibits superior computational efficiency. On the Corn dataset, it achieves runtime reductions of 8.34%, 6.96%, and 4.33% over EMD, EEMD, and VMD-based models, respectively. On the Wheat dataset, the corresponding reductions are 8.80%, 6.39%, and 4.78%.

(c) Although the EWT-MSDBO-CNN-BiLSTM-A model demonstrates slightly faster execution times—improving by 0.61% and 3.65% on the Corn and Wheat datasets respectively—it shows inferior forecasting performance. This indicates that although EWT offers modest computational advantages, it is less capable of capturing deep temporal features, thus reducing overall prediction accuracy.

(d) When compared with models employing alternative optimization algorithms (such as GA and DBO), SVMD-MSDBO-CNN-BiLSTM-A achieves substantial runtime reductions. On the Wheat dataset, it reduces time by 26.71% and 17.59% compared to SVMD-GA-CNN-BiLSTM-A and SVMD-DBO-CNN-BiLSTM-A, respectively. On the Corn dataset, the improvements are 26.72% and 17.27%, respectively. These results demonstrate the effectiveness of MSDBO in balancing forecasting accuracy and execution efficiency.

(e) Structurally simplified versions—such as SVMD-MSDBO-BiLSTM and SVMD-MSDBO-CNN-BiLSTM—achieve faster runtime, reducing execution time by 24.72% and 15.07% on the Corn dataset, respectively. However, their forecasting performance is significantly degraded, indicating that simplification comes at the cost of predictive power and limits their suitability for high-stakes applications.

### 5.6 Model interpretability analysis

To enhance the interpretability of our hybrid model in agricultural forecasting, we analyze the attention weights within the BiLSTM-Attention module. This mechanism dynamically assigns weights to input features—the original price series, decomposed Intrinsic Mode Functions (IMFs), and residual—revealing the model’s prioritization. High-frequency IMFs capture short-term noise (e.g., weather disruptions or speculation), while low-frequency IMFs and residuals reflect long-term trends, including seasonal cycles and economic influences, bridging model outputs to market drivers.

We first assess the attention weight distribution of the top-performing SVMD-MSDBO-CNN-BiLSTM-A model on corn and wheat datasets. Weights, computed as softmax-normalized averages over test samples and timesteps (summing to 1), highlight the relative importance of the original price, IMFs (from high to low frequencies), and residual. We then compare decomposition variants—EMD-MSDBO-CNN-BiLSTM-A and SVMD-MSDBO-CNN-BiLSTM-A—across datasets to evaluate how decomposition affects feature prioritization. EMD (empirical) and SVMD (variational) were chosen for their distinct approaches—EMD’s data-driven nature versus SVMD’s optimized separation—to test weight consistency robustly.

**Fig 15:**
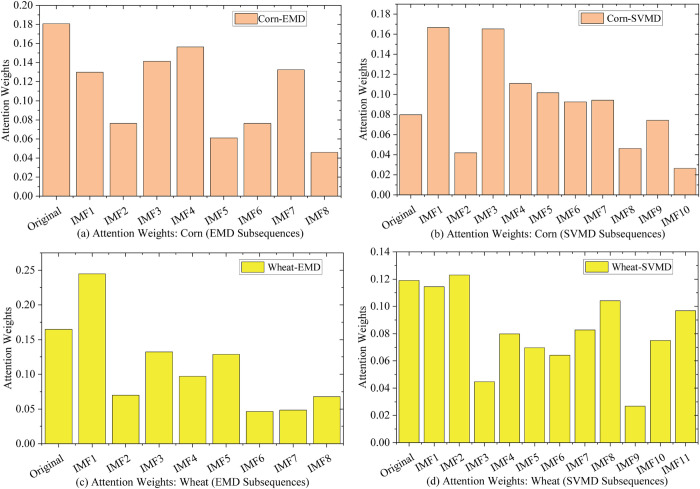
Comparative attention weights: corn and wheat with EMD and SVMD.

Fig 15 shows attention weight distributions across datasets and methods, with subplots: (a) Corn with EMD, (b) Corn with SVMD, (c) Wheat with EMD, and (d) Wheat with SVMD.

For SVMD-MSDBO-CNN-BiLSTM-A:

(a) In corn (EMD), weights are uniform, with the original price at 0.181, followed by high-frequency IMFs (e.g., IMF1 0.130, IMF3 0.141), tapering to IMF8 (0.046), indicating focus on short-term noise like weather shocks, aligning with corn’s volatility.

(b) In corn (SVMD), weights decline steeply, with original price at 0.080, peaking at IMF1 (0.167) and IMF3 (0.165), decaying to IMF10 (0.026), emphasizing noise filtering and trend shift.

(c) In wheat (EMD), decay is gradual (original 0.165, IMF1 0.245), with less high-frequency focus (e.g., IMF2 0.070), reflecting stability and trend drivers like seasonal cycles.

(d) In wheat (SVMD), weights concentrate on original price (0.119) and mid-to-low IMFs (e.g., IMF2 0.123, IMF8 0.104), with residual (IMF11 0.097), focusing on structural trends.

Comparing methods, EMD-MSDBO-CNN-BiLSTM-A shows uniform distributions: corn [(a)] from 0.181 (original) to 0.046 (IMF8); wheat [(c)] from 0.165 (original) to 0.048 (IMF8), suggesting mode mixing overemphasizes noise. SVMD-MSDBO-CNN-BiLSTM-A [(b) and (d)] exhibits steeper profiles, highlighting SVMD’s advantage in reducing aliasing and enhancing trend focus.

Across datasets, low-frequency IMFs and residuals exceed 0.4 aggregate, reinforcing trend contributions (e.g., seasonal and economic factors). Differences include corn’s high-frequency bias (volatility) versus wheat’s low-frequency dominance (stability), underscoring SVMD’s edge in volatile scenarios.

### 5.7 Practical implications

The high predictive accuracy and interpretability of the proposed SVMD-MSDBO-CNN-BiLSTM-A framework offer significant practical benefits for key stakeholders in agricultural commodity markets. The attention-based interpretability mechanism offers critical insights into the model’s decision-making process. As detailed in Sect 5.6 and illustrated in Fig 15, the framework demonstrates a distinct ability to discern and leverage market signals across different frequency domains. Specifically, it differentiates between high-frequency components such as IMF1 through IMF3, which represent short-term market noise, and low-frequency along with residual components that reflect underlying long-term trends. This capability enables tailored decision-support across sectors.

For traders and financial institutions:

(a) Short-term trading signals: the model’s facility in capturing high-frequency fluctuations (e.g., IMF1–IMF3) allows traders to detect signals from transient market disturbances, speculative activity, or sudden supply-demand shifts. Predictions coupled with elevated attention weights on these components can inform short-term trading strategies and optimize timing in futures contracts.

(b) Portfolio risk management: accurate volatility forecasts can enhance Value-at-Risk (VaR) models. Institutions can dynamically adjust hedging strategies when the model indicates heightened attention to high-frequency components, serving as an early warning for potential volatility episodes.

For Farmers and agricultural producers:

(a) Optimal sales timing: the model’s emphasis on mid- to long-term trends (e.g., low-frequency IMFs and residues) helps identify seasonal and macroeconomic patterns. As Fig 15 illustrates, the pronounced attention weight on trend components in wheat data lends reliability to medium-term forecasts, supporting decisions on selling windows and storage strategies to maximize profit.

(b) Production and inventory planning: forecasts of sustained upward trends may justify expanding cultivation acreage or investing in additional storage capacity, providing a data-driven basis for operational planning.

For policymakers and government agencies:

(a) Food security early-warning systems: the model can be integrated into early-warning systems that monitor long-term trend components. Predicting critical price inflation enables proactive measures such as strategic reserve releases or trade policy adjustments to stabilize markets.

(b) Evidence-based policy formulation: model interpretability offers quantitative evidence on drivers of price volatility. Persistent attention to high-frequency components (e.g., IMF1) suggests short-term speculative influences, calling for market oversight policies. Conversely, dominance of low-frequency drivers indicates structural or supply-side issues, warranting interventions in production support, climate adaptation, or infrastructure.

## 6 Conclusion

This study introduces a novel hybrid forecasting model for agricultural commodity prices, integrating the SVMD denoising technique, the BiLSTM deep learning method with an attention mechanism, and the improved MSDBO optimization algorithm. The BiLSTM-Attention-CNN model leverages BiLSTM units, an attention mechanism for feature prioritization, and a CNN component to capture nonlinear dynamics and temporal dependencies in daily price data for corn and wheat from the Chicago Board of Trade (CBOT), evaluated against ten benchmark configurations. The results demonstrate enhanced forecasting accuracy, surpassing existing methods, and provide valuable insights into price dynamics through the attention mechanism, supporting risk management, policy-making, and market stability in volatile global commodity markets. The key findings of this research are illustrated as follows:

(a) SVMD-MSDBO-CNN-BiLSTM-A model consistently shows lower RMSE, MAE, and MAPE values, and high *D*_*stat*_ in both corn and wheat datasets, the four values achieved on corn data are 3.5701, 2.4611, 0.6169% and 73.9097% respectively, indicating its superior forecasting capability compared to other models.

(b) On both datasets, SVMD-CNN-BiLSTM-A achieves better performance compared to SVMD-BiLSTM. On the corn data, RMSE, MAE and MAPE achieved a reduction of 21.58%, 18.58%, 17.40% and *D*_*stat*_ achieved an improvement of 2.00%. This result confirms that incorporating CNN for feature extraction and the attention mechanism for weight assignment significantly improves the model’s forecasting accuracy.

(c) Utilizing EMD, EEMD, EWT, VMD and SVMD for data decomposition, SVMD-based hybrid forecasting model (MSDBO-CNN-BiLSTM-A) achieves the best results, for example, on the wheat data, the smallest RMSE, MAE and MAPE, and the largest *D*_*stat*_ were obtained, which were 3.9701, 3.1016, 0.6377% and 79.7665%, respectively. The results show that the importance of data decomposition in enhancing forecasting accuracy.

(d) On both datasets, the MSDBO-based prediction model obtained better prediction performance. For instance, on wheat data, MAPE obtained 13.78% and 10.34% reduction, respectively. This result indicates that MSDBO outperforms both GA and DBO in optimizing BiLSTM model’s hyperparameters, demonstrating the efficacy of the improved DBO method. And furthermore, the effectiveness of optimization algorithms combined with stack-based models in price forecasting is demonstrated.

(e) Across both datasets, the MSDBO-based forecasting model consistently delivered superior predictive performance. For instance, on the wheat dataset, the MAPE was reduced by 13.78% and 10.34% compared to models optimized using GA and DBO, respectively. These results underscore the enhanced capability of MSDBO in fine-tuning the hyperparameters of BiLSTM architectures, validating the effectiveness of the improved DBO strategy. Furthermore, they highlight the strong potential of integrating advanced metaheuristic optimization techniques with stacked deep learning models for accurate and robust price forecasting.

(f) Across datasets, low-frequency IMFs and residuals contribute over 0.4 of aggregate attention weight, highlighting their role in capturing long-term trends (e.g., seasonal and macroeconomic factors). Corn shows a higher high-frequency bias (volatility adaptation), while wheat exhibits low-frequency dominance (stability), confirming SVMD’s robust interpretability under varying market conditions. This capability offers clear practical value: traders can exploit high-frequency signals for short-term strategies, farmers can rely on trend components for sales timing and production planning, and policymakers can use frequency dominance patterns to guide market stabilization and food security interventions.

While the proposed SVMD-MSDBO-CNN-BiLSTM-A model demonstrates robust performance in forecasting agricultural commodity prices, several limitations warrant consideration. The current attention mechanism provides valuable insights into feature prioritization but lacks a comprehensive quantification of interactions among predictive variables—such as market trends, seasonality, and macroeconomic indicators—or their individual contributions to price movements. Additionally, the model does not incorporate exogenous factors, such as weather conditions, geopolitical events, or global supply chain disruptions, which are critical drivers of commodity price volatility and remain unaccounted for in the present framework. Furthermore, the generalizability of the model across diverse economic systems, such as energy or metals markets, remains untested, potentially limiting its adaptability to varying market dynamics.

Future research should address these limitations to enhance model efficacy and interpretability. A detailed exploration of the relative importance of predictive features, including market trends, seasonal cycles, and macroeconomic indicators, could be pursued using advanced interpretability techniques, such as SHAP (SHapley Additive exPlanations) or LIME (Local Interpretable Model-agnostic Explanations), to disentangle their interactive effects on price fluctuations. Integrating exogenous variables—e.g., weather data, geopolitical risk indices, and supply chain metrics—into the model framework could further improve forecasting accuracy by capturing external influences. Moreover, extending the methodology to other commodities or financial instruments, such as energy or metals markets, would validate its robustness and adaptability across diverse economic contexts, broadening its practical applicability and providing a foundation for cross-market predictive analytics.
